# Incomplete homicide-suicide in Hunan China from 2010 to 2019: characteristics of surviving perpetrators

**DOI:** 10.1186/s12888-021-03574-8

**Published:** 2021-11-17

**Authors:** Qiaoling Sun, Jiansong Zhou, Huijuan Guo, Ningzhi Gou, Ruoheng Lin, Ying Huang, Weilong Guo, Xiaoping Wang

**Affiliations:** Department of Psychiatry, National Clinical Research Center for Mental Disorders, China National Technology Institute on Mental Disorder, the Second Xiangya Hospital of Central South University, Renming Road 139, Changsha, 410011 Hunan Prov. China

**Keywords:** Homicide-suicide, China, Mental disorder, Depression, Motivation

## Abstract

**Background:**

The mental and psychological conditions of the individuals involved in homicide followed by suicide (H-S) are still unclear, especially in China. Information on these aspects cannot be accurately obtained due to the death of perpetrators. This study aims to demonstrate the characteristics of incomplete H-S with surviving perpetrators, which provide the possibility to obtain detailed mental and psychological information.

**Methods:**

This study explores incomplete H-S events occurred in Hunan, China from 2010 to 2019, in which the surviving perpetrators received a forensic psychiatric assessment. Three aspects of information, i.e., the subject’s demographic, clinical and criminal information, were recorded and analysed.

**Results:**

125 incomplete H-S incidents involved 166 victims were found in the current study. A total of 112 (89.6%) perpetrators were diagnosed with mental disorders, but only 56 of them had a history of consultation due to mental problems, and only one of them adhered to treatment. In most cases, the motivation is related to the psychopathological states, with the most common diagnosis being major depression, followed by schizophrenia. Gender difference was significant among the subjects: females were more likely to have a suicidal history, to be diagnosed as major depression and to be motivated by delirious altruism and family problems.

**Conclusions:**

This study indicated that psychopathology might be a predisposing factor, which highlighted the importance of mental state assessment for the population involved in incomplete H-S. A clearer understanding of the role of mental disorders might be helpful for the intervention of H-S.

## Background

Homicide followed by suicide (H-S), which is also named “homicide-suicide” and sometimes known as “murder-suicide”, is a lethal event in which one person kills another and subsequently ends his or her own life [1]. H-S is a relatively rare incident compared to homicide or suicide alone. Studies on this topic have been carried out in many regions of the world, such as Australia [2], Oklahoma [3] and England and Wales [4, 5]. But most studies were performed in the United States [1, 6], where the incidence rate was reported to be ranged from 0.05 [5, 7] to 0.3 per 100,000 persons annually [8, 9]. Although a slightly higher rates (0.89 per 100,000) was fond in the South African [10], its incidence is still quite low. However, H-S incidents may produce long-lasting consequences for the involved families and the whole society [11], including strong emotional impact on survivors, especially when children are involved. In addition to significant financial burdens related to complex investigations of the incident, H-S also brings challenges for mental health specialists in the assessment and intervention of such events.

Theoretical studies have been focused on typology and theory of H-S. Various classification frameworks have been proposed to classify the different subtypes of H-S. In 1992, Marzuk et al. [9] proposed a typology system based on victim–perpetrator relationship and motives. In 1995, Felthous et al. [12] integrated the psychopathological factors into Marzuk et al.’s classification. In 2007, Harper et al. [13] classified the H-S on thematic context. In 2014, Joiner et al. [14] proposed the “perversion of virtue” framework, which argues that H-S is the result of primary suicidal intent that lead to the perversion of four virtues. Later, Knoll et al. [15] proposed a classification scheme includes both elements of the Marzuk typology and the Felthous methods. Most recently, Jordan et al. [16] developed an empirically based typology that consist of eight H-S subtypes. Besides, some theories have been suggested to apply to H-S, such as the strain theory, stream theory, psychodynamic theory, psycho-evolutionary theory, social integration theory, attribution theory, and masculinity theory [11, 17]. These classification frameworks and theories mainly involve factors such as motivation, relationships between perpetrators and victims, and psychopathology.

Except theoretical researches, other studies investigated the prevalence of H-S, mostly focus on the complete H-S with a successful suicide, and its related factors, and a few of them compared the profiles of H-S with either homicide only or suicide only. Generally these studies found that about 75% of H-S involved a female victim, and most perpetrators were adult males; incidents with multiple victims are also common [18–21]. 90% of the victims and perpetrators were related or known to each other, and most of them were identified as intimate partners [21]. According to a study of H-S in mainland China, an intimate relationship between the perpetrators and victims was identified in 95% cases [22]. Weapons associated with H-S incidents often included sharp instruments, blunt objects, and poisons; other methods such as strangling and drowning were also included [4]. Studies in the United States, where firearm ownership is allowed, showed that the gun was involved in the majority of deaths associated with H-S incidents [1, 3]. Through comparing H-S incidents to homicides, a study showed that when the motivation is associated with sickness, stress or possessiveness, the perpetrators are more likely to die by suicide [23].

Although we have a certain understanding of H-S, there is still little known about of why some suicide decedents killed others before death. It is generally believed that a considerable number of individuals who died by suicide after killing others are psychiatrically disturbed [24] [20]. The estimated prevalence of mental illness in different samples ranged from 20% [25] to 91% [26]. Recently, a study in England and Wales estimated that 40% of H-S perpetrators had a history of seeking medical care or consultation within a month before the incident [19]. The variation at least partly attributed to different definition of mental illness and different sources of information.

Data of H-S, especially about mental disorder, are often retrieved unsystematically due to problems in information acquisition. Most information in the existing literature come from the homicide or suicide death system, such as National Violent Death Reporting System in United States [1, 6], Office for National Statistics in England and Wales [4], and Hong Kong Police Criminal Records Bureau [22]; part of the data even came from the news [27]. Descriptive characteristics of the perpetrators have been well established by existing literature, but little is known about the mental health factors related to these incidents.

To capture psychological and contextual circumstances preceding the complete H-S with a successful suicide, the psychological autopsy [24, 28, 29] method was applied. Clinical data were collected from medical records, such as record of mental health service institutes or sometimes records from supervising clinicians responsible for the patients’ care. But, due to the lack of information and detailed personal history, the data of the perpetrators’ behavior and mental health history are prone to errors. Although the interview of relatives or close friends of the perpetrators were involved in some psychological autopsy reports, the accuracy of the information is unknown. In addition, the incidence of violent behavior or mental symptom are likely to be underestimated, as the lack of such information does not necessarily mean that the behaviors or symptoms did not occur. Due to the death of the perpetrators, it is difficult to obtain information on the mental state of the perpetrators at the time of the event. Researches showed that the rate of mental disorders varied greatly from 20 to 91% [25, 26], suggesting the difficulty to determine a diagnosis of mental illness after the perpetrator’s death. As the systematic analyses of mental illnesses are helpful for a better understanding of such events [30], a direct interview with perpetrators is needed. And the incomplete H-S events, in which the perpetrators survived, provide the possibility for research in this area.

Besides, this problem is still poorly understood in China, as H-S studies were rarely conducted in the Chinese context. Only some studied in Hong Kong (one case report [31], and two descriptive studies [22, 32]), and a research in mainland China using data from Chinese news from 2000 to 2014 [27] provided some information related to H-S. Furthermore, mental and psychological conditions related H-S are often difficult to decipher, due to the death of perpetrators. With the absence of accurate motivations and assessments of mental state, it is difficult to acquire precise and complete information of factors that contribute to the occurrence of H-S. Therefore, in order to get a better understanding of the phenomenon in China and its psychological characteristics, this study includes the incomplete H-S events occurred in Hunan, China from 2010 to 2019.

## Methods

The definition of H-S differs between studies, and the interval between the homicide and the suicide ranged from 24 h to 30 days [33, 34]. For most cases, the suicide occurred immediately after the homicide. In this study, the incomplete H-S referred to homicide or attempted homicide, followed by attempted suicide within 3 days. This study was launched by the Forensic Assessment Centre of the Second Xiangya Hospital, and was approved and supported by the other 7 qualified forensic institutions. The study protocol was approved by the Ethics Committees of the Second Xiangya Hospital; and all the researchers signed a written commitment to protect the privacy of subjects.

In China, perpetrators suspected of suffering from a significant mental disorder are mandated to undergo a forensic psychiatric assessment by qualified forensic institution [35]. There are 8 qualified forensic psychiatric institutions in Hunan province located in the south-central of China. The province’s population was enumerated at 65.701 million in 2010 and reached 69.18 million by the end of 2019. Detailed descriptions of forensic psychiatric assessment in China can be seen in previous literature [36, 37]. Usually, the assessment consists of an interview, electroencephalograph testing and intelligence quotient testing; no less than two experts to do the forensic psychiatric assessment, and the diagnoses according to the International Classification of Diseases 10 was made by these two experts. If the opinions of these two experts differ, both opinions should be recorded in the report. But in this study, we did not found any different opinion in the incomplete H-S cases.

Incomplete H-S cases were identified by scanning forensic archives of all criminal suspects who had received forensic psychiatric assessments in Hunan province, from 1 January 2010 to 31 December 2019. All the data were retrieved from the forensic archives, including the perpetrator’s demographic information, criminal files, medical records, as well as details and conclusions of the forensic psychiatric assessments. Three aspects of information, i.e., the subject’s demographic information (such as the gender, age, residence, marital status), clinical information (such as consultation history due to mental problems, history of psychiatric treatment, current diagnosis) and criminal information (age at the current crime, methods of homicide and/or suicide, number of victims, information of victims), were collected using a purpose designed standard data collection form that was specially designed for the present study.

The main findings are presented as numbers with proportions. In consideration of the obvious gender difference between homicide and suicide, we also compared the features of perpetrators between different gender. All the statistical analyses were computed with the SPSS (Version 22.0; IBM), with the significance level was set at 0.05.

## Results

### Demographics of perpetrators

A total of 125 incomplete H-S incidents were identified during the study period. The characteristics of perpetrators were presented in Tables 1 and 2. The average time interval from the incident to forensic assessment is 1.7 months. There were 76 (60.8%) male perpetrators and 49 (39.2%) female perpetrators with most of them were aged 18–49 years old; only three perpetrators were under 18 years old and one perpetrator was over 65 years old (see Table 1 for details age composition). Half of the perpetrators were married at the time of the event. The majority of perpetrators was living in rural areas and most of them had a low or medium education level. And 34 (27.2%) perpetrators were found to have a history of domestic violence. In terms of the number of victims, 94 (75.2%) cases were associated with a single victim, and 31 cases (24.8%) were associated with multiple victims, with the highest number of victims died in one single case being four.

**Table 1:** Demographic characteristics of H-S perpetrators

Variable	Total (125)				
		Male (76)	Female(49)	Statistics	P
Time interval from the incident to forensic assessment (months)	1.7 ± 2.310	2.03 ± 2.366	1.22 ± 2.153	1330.00^a^	0.005
Age					
Mean ± SD	38.86 ± 11.246	38.29 ± 12.362	34.63 ± 8.925	1.918^b^	0.058
< 18	3 (2.4)	2 (2.6)	1 (2.0)		
18–34	55 (44.0)	29 (38.2)	26 (53.2)		
35–49	49 (39.2)	30 (39.5)	19 (38.7)		
50–64	17 (13.6)	14 (18.4)	3 (6.1)		
≥ 65	1 (0.8)	1 (1.3)	0 (0.0)		
Unemployment	58 (46.4)	32 (42.1)	26 (53.1)	1.438^c^	0.230
Marital status				21.222^d^	< 0.001
Never married	29 (23.2)	27 (35.5)	2 (4.1)		
Married	83 (66.4)	40 (52.6)	43 (87.8)		
Divorced	11 (8.8)	7 (9.2)	4 (8.2)		
Unknown	2 (1.6)	2 (2.6)	0 (0.0)		
Residence				1.150^c^	0.284
Urban	39 (31.2)	21 (27.6)	18 (36.7)		
Rural	86 (68.8)	55 (72.4)	31 (63.3)		
Education^1^				2.986^d^	0.232
Low	39 (31.2)	28 (36.8)	11 (22.4)		
Medium	76 (60.8)	42 (55.3)	34 (69.4)		
High	10 (8.0)	6 (7.9)	4 (8.2)		
History of domestic violence	34 (27.2)	22 (28.9)	12 (24.5)	0.299^c^	0.585

*SD* standard deviation. Education^1^: Education level ≤ 6 years was defined as low, 6 years < education level ≤ 12 years was defined as medium, education level > 12 years or college was defined as high. ^a^: Mann-Whitney U test, ^b^: Two simple t-test, ^c^: Chi-squared test, ^d^: Fisher’s exact test

**Table 2:** Medical and criminal characteristics of H-S perpetrators

Variable	Total (125)	Male (76)	Female(49)	Statistics	P
Consultation history due to mental problems				0.173^c^	0.904
No, n (%)	69 (55.2)	41(54.9)	28 (57.1)		
Psychiatry Department, n (%)	39 (31.2)	24 (31.6)	15 (30.6)		
Other departments, n (%)	17 (13.6)	11 (14.5)	6 (12.2)		
Mental history, n (%)	55 (44.0)	35 (46.1)	20 (40.8)	1.062^c^	0.745
Drug treatment experience				2.661^d^	0.209
No, n (%)	79 (63.2)	44 (57.9)	35 (71.4)		
Irregular or stop taking the drugs, n (%)	45 (36.0)	31 (40.8)	14 (28.6)		
Regular medication, n (%)	1 (0.8)	1 (1.3)	0 (0.0)		
Suicide history				16.165^c^	< 0.001
Attempted suicide, n (%)	22 (17.6)	12 (15.8)	10 (20.4)		
Suicide idea, n (%)	36 (28.8)	13 (17.1)	23 (46.9)		
No, n (%)	67 (53.6)	51 (67.1)	16 (32.7)		
Current diagnoses				20.411^d^	0.001
Organic mental disorders	9 (7.2)	7 (9.2)	2 (4.1)		
Schizophrenia	31 (24.8)	26 (34.2)	5 (10.2)		
Major depression	52 (41.6)	22 (28.9)	30 (61.2)		
Bipolar disorder	10 (8.0)	4 (5.3)	6 (12.2)		
Other^2^	10 (8.0)	6 (7.9)	4 (8.2)		
Suicide method				6.861^d^	0.229
Cutting/stabbing	43 (34.4)	30 (39.5)	13 (26.5)		
Self-poisoning	26 (20.8)	15 (19.7)	11 (22.4)		
Drowning	15 (12.0)	5 (6.6)	10 (20.4)		
running head against the wall/jumping off a building	11 (8.8)	8 (10.5)	3 (6.1)		
Surrendered or Be stopped	16 (12.8)	10 (13.2)	6 (12.2)		
Other^3^	14 (11.2)	8 (10.5)	6 (12.2)		
Motivations of incomplete H-S				59.657^c^	< 0.001
Delirious altruism	38 (30.4)	5 (6.6)	33 (67.3)		
Hallucination/delusion	29 (23.2)	26 (34.2)	3 (6.1)		
Related with other mental states/Perpetrators’ primary suicide ideation	14 (11.2)	12 (10.5)	2 (4.1)		
Relationship breakdown/Anger after conflict/No specified	15 (12.0)	15 (19.7)	0 (0.0)		
Family problems	29 (23.2)	18 (23.7)	11 (22.4)		
Circumstance associated with the HS				2.517^d^	0.485
Financial problem	18 (14.4)	13 (17.1)	5 (10.2)		
Other relationship problem	43 (34.4)	24 (31.6)	19 (38.8)		
Physical health problem	7 (5.6)	3 (3.9)	4 (8.2)		
Victims killed in an incident				1.710^d^	0.458
1	94 (75.2)	54 (71.1)	40 (81.6)		
2	23 (18.4)	16 (21.1)	7 (14.3)		
≥ 3	8 (6.4)	6 (7.8)	2 (4.1)		

^c^: Chi-squared test, ^d^: Fisher’s exact test

There are significant differences in the time interval from the incident to forensic assessment and marital status between genders; females were associated with significantly shorter time interval from the incident to forensic assessment and were more likely to be married at the time of the incident.

### Clinical characteristics of perpetrators

Of all the perpetrators, 44.8% had a history of consultation due to mental health problems. Most of them (39 in 56) visited the psychiatry department at a hospital, some visited other departments, such as the neurology department. Of them 44.0% perpetrators had been diagnosed with mental disorders, but only 36.8% received the drug treatment and only 1 perpetrator continued receiving medication. With regard to the suicidal issue, 17.6% of the perpetrators had a history of attempted suicide and 28.8% had suicidal ideation. There was a significant difference in the suicidal history between genders, with females being more likely to have a history related to suicide. Among the female subjects, 67.3% females had a history of suicide, the percentage was only 32.9% for males.

There were 112 (89.6%) perpetrators diagnosed as mental disorders, with most common diagnosis being major depression, followed by schizophrenia. Most perpetrators with bipolar disorder (9/10) were in a depressive episode. Two perpetrators (1 male, 1 female) were diagnosed as mental disorders due to use of psychoactive substances, 2 male perpetrators were diagnosed as acute and transient psychosis, 2 female perpetrators were diagnosed as stress-related disorders, 1 male perpetrator was diagnosed as hysteria, 1 male perpetrator was diagnosed as personality disorder, and 2 perpetrators (1 male, 1 female) were diagnosed as mental retardation. There was a significant difference in the current diagnosis between genders. Females were more likely to be diagnosed with major depression (61.2%), while males were more likely to be diagnosed with schizophrenia (34.2%) and major depression (28.9%).

### Methods of homicide

The most common weapons of homicide were sharp instruments, which were related to 83 victims (50%). This is followed by methods of suffocation/carbon monoxide (17.5%) and blunt instruments/falls (14.5%). Other methods, such as drowning and poisoning were also involved. In 2 cases, firearm was used, involving 6 victims and the death of 2 perpetrators.

### Methods of attempted suicide

The most common methods of suicide were cutting/stabbing (34.4%), self-poisoning (20.8%) and drowning (12.0%). Ten perpetrators (6 males, 4 females) surrendered and demanded death penalty, and 6 perpetrators (4 males) were stopped before committing suicide. Other methods, such as running head against the wall/jumping off a building (11, 8.8%), hanging (1 male, 2 females), carbon monoxide (1 male, 2 females), self-burning (1 male) and swallowing a light bulb (1 female) were also involved in some cases. Suicide methods in 4 cases were not recorded.

### Clinical and psychosocial factors related to incomplete H-S

The most important motive for perpetrators to execute incomplete H-S was delirious altruism. Thirty-three mothers and two fathers killed their young children, and 3 adult males killed their mother. Twenty-nine perpetrators were motivated by hallucinations/delusional symptoms and most of them (21) was diagnosed with schizophrenia. Eight incomplete H-S cases were driven by perpetrators’ primary suicide ideation. In 5 cases, perpetrators and victims agreed to die together and the victims were willing to be killed; and in 3 cases, the perpetrators wanted to end their lives by killing innocent people randomly and being shot by legal force. There were still 6 cases caused by other mental issues, of which 5 perpetrators (2 with schizophrenia, 1 with major depression with psychotic symptoms, 1 with acute and transient psychosis, and 1 with mental disorder due to use of psychoactive substances) were in a state of disintegrated state when they committed incomplete H-S, with no clear motive found afterwards. And 1 perpetrator with mental disorder caused by epilepsy was in a state of hazy consciousness and was unable to recall the course afterwards.

Motivations that were not related to mental disorders include relationship breakdown, anger after conflict and family problems. Four male perpetrators killed their girlfriends or spouses because of a broken relationship, 9 male perpetrators were motivated by anger after conflict with the victims, and 29 (23.2%) perpetrators killed their family members due to family issues. Motivations in 2 cases were not specified. Among the 2 cases, one perpetrator with depression killed another patient because he did not want to be hospitalized, and then attemptted suicide under the influence of depressive symptoms. In another case, with the thought that he, as a patient with epilepsy, and his eldest son who also suffered from epilepsy, were the burden of his younger son, thus, he killed his eldest son and then attemptted suicide.

There was a significant gender difference in motivation. Females were more likely to be motivated by delirious altruism (67.3%) and family problems (22.4%), while males were more likely to be motivated by hallucination/delusion (34.2%) and family problems (23.7%).

As is shown in Table 2, other specific situational factors associated with suicide following homicide were recent relationship problems (34.4%), job or financial problems (14.4%) and physical health problems (5.6%).

### Characteristics of victims

There were 166 victims in total, of whom 130 people died. The number of male victims and female victims were almost equal. The distribution of the relationship between victims and perpetrators is shown in Table 3. The majority of victims were known to the perpetrator, with some of them being the intimate partner relationship. The victims were the perpetrators’ children in 63 (38.0%) cases, their parents in 16 (9.6%) cases, and their spouses or partners in 24 (14.4%) cases. The perpetrators were strangers to the victims in only 8 (4.8%) cases.

**Table 3:** Characteristics of H-S victims

Variable	Number (166)	%
Relationship to perpetrator		
Child	63	38.0
Parent	16	9.6
Spouse / boyfriend / girlfriend	24	14.4
Other family	16	9.6
Acquaintance	39	24.5
Stanger	8	4.8
Killing methods		
Suffocation/carbon monoxide	29	17.5
Drowning	12	7.2
Sharp instrument	83	50.0
Blunt instrument/falls	24	14.5
Poisoning	9	5.4
Firearm	6	3.6
Unknown	3	1.8
Death	130	78.3
Male	87	52.4
Female	79	47.6

## Discussion

To our knowledge, this is the first study on the characteristics of incomplete H-S, in which the perpetrator survived and received a forensic psychiatric assessment in mainland China. We focused on gender differences, psychosocial and clinical information, hoping these aspects of incomplete H-S would help in the identification and prevention of individuals at risk and to some extent, improve our understanding of H-S.

Most of the perpetrators in incomplete H-S were males, which is consist with previous studies about H-S. However, the male-female ratio is not as great as those found in previous H-S studies. For example, 95% H-S perpetrators were men in the first study of H-S in mainland China [27], while a study in Hong Kong showed that 68.7% of H-S perpetrators were male [32]. As for the gender ratio of victims in incomplete H-S, we have found slightly more males than females, which is inconsistent with previous results in H-S. In a study in mainland China, Densley reported that 50% of the victims in H-S were female, compared to 33% of males and 17% of cases where the gender of the victims was unknown [27]. A study in Hong Kong reported that 64% of the victims in H-S were female [22]. The most likely explanation for the variation is that the cases in our study are incomplete H-S incidents with surviving perpetrators, while cases in previous studies are H-S incidents include both complete and incomplete cases. The present study is based on individuals who had undergone forensic psychiatric assessment and it is possible that more male perpetrators had died, leaving them no chance to receive an assessment.

Analysis of the data reveals significant gender differences in perpetrators’ suicide history, current diagnosis and motivation. Females showed a higher rate of suicide history, they were more likely to be diagnosed as depression and often motivated by delirious altruism, the idea of saving their children from the horrible fate or future [38]. Sometimes these cases are understood as “extended suicides” as the depressive mothers have also been suggested to consider their children as their continuity, and then they naturally end their children’s lives when they suicide due to the unworthy existence [9]. It suggested that the incomplete H-S in woman might be more related to depression and suicide ideation, which implied that public health measures aimed at reducing suicide might be helpful to prevent such events.

The majority of studies have indicated that psychopathology is a predisposing factor of H-S. H-S perpetrators are more likely to suffering from mental illness, with the most common disease being depressive disorder, followed by psychosis [39]. A study in England and Wales found that 62% perpetrators had mental health problems, with the most common diagnosis of depression, but few had received any mental health services recently before the incident [19]. Furthermore, depression was found to be more common in female perpetrators who killed their children [40]. In addition to depression, individuals with psychosis, especially schizophrenia, might be at higher risk for violence, including H-S, as their current delusion was associated with aggression [41].

As described in this study, the mental illnesses leading to hallucinations, delusions or depression, may well have been a factor in some incomplete H-S incidents. However, the path from mental disorders to it might not be a straight line, and mental disorders are often a part of the complex story and can be affected by a variety of other factors, such as treatment. In this study, 110 perpetrators were diagnosed as mental disorders, but only 56 had a history of consultation due to mental health issues and only one of them continued treatment. Undetected and untreated psychosis may be more closely related to incomplete H-S. Many studies have reported that most of the aggressive behaviors of individuals with psychosis occurred during the first episode of the illness [42] or before contacting the mental health system [43]. Compared with the later stage, the relationship between aggressive and psychosis is the most prominent during the first episode [44]. Treatment compliance is also considered to be closely related to aggressive behavior. A meta-analysis on first-episode psychosis showed that involuntary treatment after the treatment regimen started was closely related to aggressive behavior [42]. Similarly, non-compliance to treatment can also lead to serious aggressive behaviors. This suggests that early diagnosis of diseases and effective, long-term treatment should be highly valued.

Although the rate of mental illness is high in the subjects included in this study, it does not mean that mental disorders will lead to or significantly increase the risk for incomplete H-S; it implies that it is necessary to assess the mental state in incomplete H-S population and to improve our understanding of the role of mental illnesses, although the degree of impact of mental illnesses on such events are needed to be determined. In most incomplete H-S cases, perpetrators are required to undergo a psychiatric assessment to determine whether they were mentally abnormal at the time of the event. However, such a process cannot be completed if the perpetrators have died of suicide.

In this study, apart from the events with mental disorder-related factors, most cases were motivated by family problems. Furthermore, situational factors, including recent relationship problems, job or financial problems and physical health problems, were also associated with the incomplete H-S. This is consistent with previous studies which tried to elucidate the circumstances under which the events occurs [6, 45]. A study showed that H-S typically occurs when noxious stimuli were present, such as financial or family problems [34]. Major changes in social environment are also influencing factors. Failure in achieving highly valued goals, such as being rejected by an intimate partner, is generally considered as a contributing factor to H-S. H-S researches showed that the perpetrator-victim relationship is typically close. Logan [21] found over half of perpetrators (53.9%) in the subjects experienced conflicts with their intimate partner.

The major limitation of this study is that we just included the incomplete H-S cases. Due to the lack of completed H-S cases, the characteristics of the study sample may have deviated from those of whole H-S sample. And the group of subjects is not representative of the general population, hence the results cannot be generalized and are only suggestive of possible associations. However, a greater understanding of the reasons for incomplete H-S may lead us to ways to reduce these catastrophic events. Additionally, the data does not represent all the incidents in Hunan, China from 2010 to 2019. Cases in which the perpetrator has not been sent for assessment are not included; however, this study has provided more accurate information of H-S, especially on psychosocial and clinical information.

## Conclusions

In summary, this study demonstrates the characteristics of surviving perpetrators in incomplete H-S events. The findings suggest that psychopathology might be a predisposing factor underlying incomplete H-S and highlight the importance of mental state assessment for the population involved in such events. Further, a better understanding of the role of mental disorders could be useful in developing appropriate interventions.

## Data Availability

The data that support the findings of this study are available on reasonable request from the corresponding author.

## Abbreviation

H-S: Homicide followed by suicide
